# Supplementary material for: A potential relationship between soil disinfestation efficacy and leaf green reflectance
Source: PLoS One. 2022 Jul 22;17(7):e0271677. doi: 10.1371/journal.pone.0271677 (PMC9307191; doi:10.1371/journal.pone.0271677)
Supplement: S1 Data — (PDF) [file pone.0271677.s001.pdf]

Viability of purslane seed, nutsedge tubers, and *Verticillium dahliae*.

| Treatment | Purslane | | Nutsedge | *V. dahliae* | |
|---|---|---|---|---|---|
| | 2018-19 | 2019-20 | 2019-20 | 2018-19 | 2019-20 |
| | % Viable | | | Microsclerotia $g^{-1}$ | |
| Non-treated control | 77.5 b[z] | 47.3 b | 83.5 b | 687.7 b | 220.0 b |
| MSM alone | 85.5 b | 59.3 c | 77.0 b | 552.2 b | 177.8 b |
| Steam alone | 3.0 a | 0.0 a | 7.0 a | 1.2 a | 0.3 a |
| Steam + MSM | 0.5 a | 0.0 a | 0.0 a | 0.2 a | 0.0 a |
| Chloropicrin | 2.1 a | 0.0 a | 1.5 a | 0.2 a | 2.5 a |

[Z]Mean separation by Duncun's multiple range test.

*Pythium ultimum* levels before and a week after treatment.

| Treatment | 2018-19 | | | 2019-20 | | |
|---|---|---|---|---|---|---|
| | Pre-treatment | Post-treatment | % Reduction | Pre-treatment | Post-treatment | % Reduction |
| | -Propagules (n $g^{-1}$)- | | | -Propagules (n $g^{-1}$)- | | |
| Non-treated control | 26.0 | 27.7 b[z] | -7 | 3.7 | 3.0 | 19 |
| MSM alone | 26.7 | 30.0 b | -12 | 1.7 | 1.7 | 0 |
| Steam alone | 32.0 | 0.0 a | 100 | 2.7 | 0.0 | 100 |
| Steam + MSM | 47.7 | 0.0 a | 100 | 2.3 | 0.0 | 100 |
| Chloropicrin | 29.3 | 0.7 a | 98 | 2.0 | 0.0 | 100 |

[Z]Mean separation by Duncun's multiple range test.

## The cumulative season-long weed densities and biomass.

| Treatment | Weed densities | | Fresh weight | |
|---|---|---|---|---|
| | 2018-19 | 2019-20 | 2018-19 | 2019-20 |
| | No. $m^{-2}$ | | g $m^{-2}$ | |
| Non-treated control | 1.38 bc[z] | 2.42 b | 4.44 ab | 5.60 b |
| MSM alone | 1.53 c | 0.85 a | 8.55 b | 2.49 a |
| Steam alone | 0.75 a | 0.08 a | 1.95 a | 0.28 a |
| Steam + MSM | 1.16 ab | 0.11 a | 3.49 a | 1.26 a |
| Chloropicrin | 0.99 a | 0.06 a | 4.19 a | 0.10 a |

[Z]Mean separation by Duncun's multiple range test.
