# Supplementary material for: A potential relationship between soil disinfestation efficacy and leaf green reflectance
Source: PLoS One. 2022 Jul 22;17(7):e0271677. doi: 10.1371/journal.pone.0271677 (PMC9307191; doi:10.1371/journal.pone.0271677)
Supplement: Attachment — Submitted filename: Response to Reviewers.docx [file pone.0271677.s002.docx]

Thank you for your time and review. The authors generally agree with your comments, and addressing your comments will improve our manuscript. Below are responses to your comments.

| Reviewer’s comment | response |
| --- | --- |
| The treatments applied for the year 2019-2020 and 2020-2021 were significantly different. Under such a situation, can these be compared? This is clearly visible from Fig. 3. In year 2019-2020, Chloropicrin and steam treatments were almost similar while it was different in 2020-2021. | Thank you for your comment. Though the treatment applications were not identical, we observed similar trends between the two seasons. In Fig 3., the locations (centers) of the distributions have the same order. In addition, Figs 2, 5, 6, and 7 show similar trends between S and P which are distinguishable from C (The yields both years followed the same trends.). Therefore, the overall effect of S and C on the plant health (observed by the colors) and the productivity are similar. Here is another important point that we needed to include in the discussion. We are aware that the both trials, the difference of the soil temperature in the steam treatments were caused by the difference of the steam machines (Fig. 1). This difference of the soil temperature may be the cause of the different results between the two trials, and there are many previous studies proving this.  This inserted into the discussion (Line 215). |
| The classification of RGB codes in the order from dark to light green seems quite arbitrary. It should be concrete based on solid statistics or physics principle. | Our choice was based on statistics (i.e., calculated from the data). In general, based upon our knowledge, there are no definite classifications of “dark green” and “light green,” and there are a number of (complicated) ways to combine R, G, and B for color analysis (e.g., Angulo & Serra, 2007). Instead of using the complicated formulas, which were not helpful in our study, we matched observed images and distributions of RGB codes. While green colors have the common property of G > R and G > B, a green color tends to be darker when (R + B) / G is close to zero (i.e., R/G and B/G are close to zero). It is statically shown in Fig 3, and the observed pattern (the order) is consistent between the two seasons. We added this explanation in the section of statistical method (Line 118) and in the section of results (Line 155). |
| L27: “We hypothesized that plant health could be quantified and treatments differentiated” to “We hypothesized that plant health could be quantified and treatments can be differentiated” | Line 27 Revised as suggested. |
| L64-66: Delete “Nitrogen and magnesium deficiencies in strawberry plants results in low chlorophyll concentration in leaves [12]” | Deleted as suggested. |
| L82: Provide the full form of “OM” | Line 81 OM was changed to organic matter. |
| L87: Was “Chloropicrin” sprayed on bare soil? | Line 87 Chloropicrin was applied at 24 mL m^-2^. by subsurface drip irrigation. |
| L98: “(PicClor 60 EC) was applied October 24, 2020” to “(PicClor 60 EC) was applied on October 24, 2020” | Line 97 Revised as suggested. |
| L110: What do you mean by RGB codes and area occupied by RGB code? Please describe in detail. | Line 109 ‘The image analysis program outputs RGB codes per image file and areas (pixels) occupied by each RGB code.’ to ‘The image analysis program outputs RGB codes and the number of pixels.’ |
| L126: Was the weight calculated for each color or only for green? | The weights were only for green, and we added one sentence (Line 130). Thank you for helping us making the manuscript clearer. |
| L141: Which packages were used in R software? Test the normality of the ratio and log transformed ratio. | lmer and lmerTest packages were used for the mixed-effects model (Bates et al., 2015; Kuznetsova et al., 2017), and we added the related references. Regarding the normality, we tested the normality of the log-transformed ratio because it is what we used in the analysis. We did not observe severe violations of normality. The p-values calculated from the Shapiro test were 0.23 and 0.42 for the 2020 data and the 2021 data, respectively, and we reported in the results section (Line 146). Thank you. |
| L192: “correlation coefficients” to “coefficient of determination” | Line 205 Revised as suggested. |
| L193: “correlation coefficients” to “coefficient of determination” | Line 206 Revised as suggested. |
| L202: “Soil pests” to “Soil borne pests” | Line 221 Revised as suggested. |
| L206-207: “weeds and hand removal is expensive.” To “weeds. Hand removal of weeds is expensive.” | Line 225 Revised as suggested. |
| L211: “environmentally friendly” to “environment friendly” | Line 230 Revised as suggested. |
| L226-227: Sentence is incomplete | Line 248 Revised as suggested. ‘Our method avoided this problem by focusing on the degree of greenness which did not require a fixed distance from the leaf to measure determine plant health.’ |
| L238-242: Meaningless, please rewrite. | We totally agree with you and deleted the text. |
| L247-248: “effective of a method of soil disinfestation as the chloropicrin application.” To “effective as the chloropicrin application for soil disinfestation.” | Line 265 Revised as suggested. |
| L250: “treasures” to “treatments” | Line 269 Revised as suggested. |
| L253: For which treatment? | Line 272 Steam treatment |
